# Impact of Public Risk Perception in China on the Intention to Use Sports APPs during COVID-19 Pandemic

**DOI:** 10.3390/ijerph191911915

**Published:** 2022-09-21

**Authors:** Peng Gu, Hao Zhang, Zeheng Liang, Dazhi Zhang

**Affiliations:** 1 School of Communication, Soochow University, Suzhou 215031, China; 2 School of Physical Education and Sports, Soochow University, Suzhou 215031, China

**Keywords:** sports APPs, risk perception theory, COVID-19, social norms, public health

## Abstract

At the onset of the 2019 coronavirus (COVID-19) pandemic, China effectively reduced the risk of a major outbreak through measures such as lockdown, quarantine and closure, which also brought the country to a standstill with normal social operations largely becoming stagnant, including suspension of production, schools and business. In active response to this non-normality, the nation has resorted to various apps to promptly restore social operations, forming a new norm of ‘offline life’ as supplementary to ‘online life’. Although a variety of increasingly sophisticated APPs have gradually restored the public’s life and work, the people’s emotions and psychology are still under influence from the risk environment of COVID-19 with high mortality and infection rates. Then, given that existing APPs have been proved effective in many areas in a risky society, is the Chinese public willing to use sports APPs to exercise as an active response to the pandemic? With risk perception theories as the foundation, this study explores the impact of risk perception on the intention to use sports apps among the Chinese public, and introduces ‘self-efficacy’ and ‘social norms’ as mediating and moderating variables, respectively; the two factors, deemed closely related to app use behaviours, have been customarily considered in previous studies. This study aims to fill the research gap in terms of the influence of risk perception on public behaviour in the context of emerging life states during global public health events, and to enrich the spectrum of risk perception theories. During the study, 1366 valid questionnaires were collected and analysed using hierarchical linear regression (HLR). The results show that risk perception, self-efficacy and social norms significantly influence the intention to use sports apps, and that the stronger the perception of the risk is, the higher the usage intention. Among the three factors, social norms during COVID-19 play a moderating role in the relationship between risk perception and the intention to use such apps.

## 1. Introduction

Since the emergence of COVID-19 in 2019, there have been 499 million cumulative infections and more than 6.18 million cumulative deaths globally [1], and these data continue to rise worldwide. To reduce deaths and infections and to safeguard people’s lives and health, many countries adopted lockdown and restriction measures (e.g., implementing closure and quarantine policies, closing gyms, restricting outdoor gatherings, and working from home) and public life and work was shut down to a certain extent [2]. Under such a circumstance, it is evident around the globe that physical activity (PA) among people has dropped considerably [3]. It may then be asked: how can people return to or maintain a level of PA when the original conditions for exercise are not possible? The same question has long been debated in the field of education. In the spring semester of 2020, after the outbreak of the pandemic, the education sector was able to resume teaching and learning activities by virtue of ‘cloud classes’, supported in large part by mobile apps and resulting in relatively satisfactory outcomes. Such evidence has proven the effectiveness of using diverse apps to facilitate work, life and other activities in the context of new social norms under epidemic containment. In this light, a further question is proposed: is the public in China willing to use sports apps to maintain or restart PA as an important part of daily life?

An app is “a software application usually designed to run on smartphones or tablet devices and provide a convenient means for the user to perform certain tasks” [4]. Statistics show that mobile device users worldwide total 7.1 billion as of 2021, and the number in 2025 is estimated to reach 7.49 billion [5]. One study found that users of smartphones spend 90% of their time on apps [6], and it is reported in another study that 49% of users open apps at least 11 times a day, with 21% of millennials doing it at least 50 times a day [7]. Of all app categories, health, fitness and sports apps account for 5.18% of the market [8], and a survey suggests that 35% use sports and fitness apps daily and 40% do so several times a week [9].

Data from the QuestMobile website show that, as of February 2021, there were more than 780 million online sports app users in China, and the number of users of specialized fitness apps was approaching 60 million [10]. During COVID-19, lockdown policies decreased people’s PA level, and sports apps provided an efficient solution to this issue [11]. During the pandemic, APPs have been emerging for the public to use that can help to resume normal social activities and facilitate online education, remote communication, pandemic monitoring and so forth. Under such circumstances, Chinese society has formed a new “online + offline” lifestyle, and the use of APPs has gradually become a key means to shape public behaviour. Therefore, investigating the public’s intention to use sports APPs can provide important reference for further improvement and development of APPs and throw light on the prospects of lifestyles based on digital technology.

Risk perception (RP) refers to an individual’s sense and evaluation of risk events and their consequences, as well as the psychological process where people make judgements about potential or possible risks and behave accordingly [12]. Existing research has demonstrated that public perceptions of the pandemic risks appreciably influence people’s subsequent behavioural decisions. In the face of the outbreak, policies of closure, daily updates on the number of infections and continual media coverage of the pandemic reinforced the public’s perception of the risk of the global health emergency and therefore every behaviour they adopted was under the influence of such factors, including using sports apps to maintain PA. Many scholars have affirmed the importance of risk perception about COVID-19. Bee and colleagues indicate that public RP of the outbreak affected attitudes towards tourism and triggered short-term travel avoidance behaviours [13]. Similarly, one study declared that public perception of risk during the pandemic was an important factor affecting the public’s adoption of protective action recommendations (PARs) [14].

Typically, previous studies on public RP and the intention to use sports APPs have measured RP variables in the context of a normative environment. A study indicates that, with public scepticism about digital security and increasing awareness of privacy, actual usage behaviour toward sports APPs is negatively influenced by user risk perceptions [15]. Schnall et al. report that health APPs trigger concerns regarding privacy and security risks and that risk perceptions positively influence people’s behavioural intentions to use mobile health (mHealth) APPs [16]. These findings have effectively validated the association between risk perception and the public’s intention to use sports APPs in the context of a normative society, providing a heuristic basis for this study.

This study uses risk perception theories to explore how RP affects the public’s intention to use sports apps in light of the new living environment under the containment of COVID-19. The public’s access to workout resources has been under restrictions due to policies of closure and quarantine during the pandemic, when external supports that they were once accustomed to have been forced to a halt. Worse still, mortality rates, high infection rates and intensive and frequent testing have combined to ignite the public’s anxiety and fear, and consequently individuals’ self-assessment is likely to have distinct changes. Existing research has established that self-efficacy (SE) is a subject’s intrinsic perception of usefulness and that SE determines an individual’s willingness to act. In this light, this study takes SE as a mediating variable. In addition, considering that social norms (SNs) play an equally important role in an individual’s behavioural intentions, SNs are included as a moderating variable to investigate the effect of public perception of risk on their intention to use sports apps in the context of COVID-19.

Special attention needs to be drawn to the fact that differences in cultural and social norms between countries are key factors influencing people’s behaviour, and this contributes to dissimilar risk perceptions and understanding of epidemics among people from different cultural backgrounds [17]; hence the unique significance of this study. Research has shown that Asian countries tend to implement stricter regulations and policies, due to their tight-knit cultures, than European countries with cultural looseness, where people have only been advised to stay at home [18]. It is thus evident that the social environment and cultural values make a difference to people’s attitudes towards the pandemic and also have a varying degree of impact on people’s psychological perception of risk. A cross-cultural study compares the relationship between public RP and cultural orientation during COVID-19 in China and the United States. The results suggest that the Chinese sample reports higher severity in RP than the US counterpart, and that different RP variables may play different roles in a certain cultural context; it is thus concluded that all differences in RP between countries are rooted in cultural context [19].

An interesting finding better illustrates the practical relevance of this study. According to statistics, in February 2020, the active number of users of China’s sports and fitness app industry soared to 89.28 million, representing an increase of 93.3% year-on-year, and the corresponding number of health management apps grew 152.8% year-on-year [20]. The link between the rapid growth in the use of sports apps and the public RP that accompanies the outbreak has raised concerns. It therefore justifies the assumption that in the early stages of a public health emergency, because of its prevalence and hazards, it is apt to trigger public panic, thus reinforcing the public’s awareness of perceived risks and prompting people to translate this awareness into actual behavioural intentions. Therefore, it is significant to explore the impact of the public’s RP on their intention to use sports apps with the new way of life taken into account as the background, filling the research gap with regard to the intention to use sports apps and casting light on RP studies about the pandemic around the world.

## 2. Literature Review

### 2.1. Pandemic Risk Perception and Health-Related Behaviour

Risk perception theories date back to the 1960s, and during that earliest stage a key paper by Starr defined risk perception as people’s reactions to events that might occur. Starr discovered that RP abilities are not only related to people’s assessment of the risk per se, but also relevant to their subjective willingness (e.g., voluntariness), and this revelation has provided a theoretical basis for subsequent RP research [21]. RP models are based on psychometric paradigms and are centred on developing a system for understanding and predicting how people react when faced with risks, such as why people are extremely averse to the risks posed by some disasters and relatively indifferent to some others [22]. To achieve this goal, researchers most commonly quantify people’s attitudes and perceptions of risk so that they can effectively judge known and unknown risks as well as risk levels of various hazards. Slove and colleagues used a psychometric paradigm model to measure RP in different dimensions, based mainly on two principles: risk perception is quantifiable and predictable; and ‘risk’ has a unique meaning for each individual [12]. Based on the above research, Langford and colleagues proposed a conceptual model containing multiple factors illustrating changes in RP. The ultimate goal is to acquire survey results of public RP, thus building a dynamic RP measuring model [23].

In past studies, risk perception has been widely applied to different disciplines, and understood differently among them [24]. For individuals, events never experienced are more likely to initiate RP, and people may have different behaviours and decisions when the risks are perceived [25]. Carman and Kooreman have measured the impact of personal RP on the willingness to get vaccinated against influenza, finding that RP plays a significant role in decisions to take action to reduce risk [26]. RP influences health-and safety-related behaviours, as demonstrated in studies on specific activities such as disease screening and vaccination. Therefore, RP is an important intervening variable in behavioural intentions.

As a worldwide public health emergency, the COVID-19 pandemic has brought new characteristics to RP, and the public’s understanding and awareness of RP is constantly being strengthened. In the context of the pandemic, Vieira and colleagues have specifically proposed an RP scale to measure the level of RP in the general public by determining fear of risks and personal psychological emotions [27]. Studies have shown that chronic negative emotions may weaken people’s immunity and disrupt the balance of physiological mechanisms. Stricken by public health emergencies, people develop more negative emotions because of unknown risks, which in turn has the potential to influence people’s judgement and perceptions [28]. Dionne and colleagues found that RP plays a decisive role in whether individuals would take protective measures during the pandemic [29]. Beldad and colleagues investigated the relationship between RP and usage continuance intention among users of Runtastic, the most popular fitness app in Germany, and it was concluded that there was no significant effect of privacy risk perception on the intention to continue using the app [30]. Studies on public RP and sports app usage intentions usually choose to measure RP variables in a normative setting with few having studied the correlation in the context of public health emergencies.

In this light, the paper aims to fill the gap in previous research by exploring the impact of the Chinese population’s RP on their subsequent intention to engage in relevant behaviours in the new social state created under lockdown polices in response to the public health emergency. Risk perception related to the COVID-19 pandemic this paper focuses on refers to people’s subjective perceptions of anxiety about the possibility of contracting the disease and concerns about their own health [31], and subsequent decision-making of adopting targeted behaviours. From the perspective of individual behavioural intentions, since personal awareness plays an important role in health behaviour, it is hypothesised that the perceived risk of outbreak will motivate the Chinese population to make behavioural decisions to stay physically active in relatively isolated spaces and also to actively take advantage of sports apps to monitor the effectiveness of their PA. Based on former study results, this study proposes the following hypothesis:

**H1.**  *Risk perception about COVID-19 is effectively predictive of the public’s intention to use sports apps.*

### 2.2. Self-Efficacy (SE) and Sports Apps

Self-efficacy (SE) describes a person’s perception of his or her behavioural capability, a concept that has been shown to be a direct predictor of health behaviours [32]. People’s acceptance of things often depends on their own understanding and perceptions, and based on their SE, people will first form expectations of the necessity and usefulness of things according to their own behavioural experience, which will then influence their decisions; thus, self-efficacy is often considered an effective predictor of the intention to use new technologies and their actual use [33]. In the sports domain, Ceasar et al. have reported the effect of promoting the benefits of PA via sports APPs in altering users’ behaviours by increasing their SE, and they conclude that it is also possible that the users quit using a particular APP if they find the disadvantages of using it outweigh the advantages, even though the APPs are somewhat beneficial to them [34]. To put it simply, when the users’ SE is higher, they will perceive greater usefulness of sports APPs, and the more ready and willing they are to engage in health-related activities. Thus, user SE positively influences the usage intention regarding sports APPs [35].

The measurement of self-efficacy can happen in a variety of groups and domains; specifically, our study measures the public’s self-efficacy perceived in the use of sports APPs, aiming to identify, through the analysis in the user group surveyed, the perceived ability that influences the continuous intention to use the APPs. When users believe in their ability to achieve certain goals by using sports APPs in one way or the other, they will choose to continue the use of the APPs; self-efficacy is influential in the usage intention towards sports APPs, and such changes take place mainly according to individual perceptions. With the above considerations, this paper hypothesises that users predict the necessity and usefulness of sports APPs based on their own behavioural experience, and when they feel fully confident in the good the APPs can do to improve physical health, their trust in the APPs is higher, which leads to greater likelihood for them to turn to sports APPs and to more actual use [36]. Previous research has shown that perceived threat, barriers, and other SE-related elements are key factors in individuals’ engagement in and continuance of pro-health behaviours [37]. During COVID-19, public SE works on the premise that people perceive that the pandemic is likely to have serious consequences for individuals, and meanwhile, they are aware of the health benefits of using sports apps and are responsive to the disease. In the light of previous research results, the mediating variable of SE is introduced in this study, and it is assumed that the intention to use sports apps is directly related to users’ desire to improve physical health, and that people with stronger SE are more willing to use sports apps. On this premise, the following hypotheses are proposed:

**H2.**  *Self-efficacy plays a mediating role between COVID-19 risk perception and the intention to use sports apps.*

**H3.**  *Due to the impact of the COVID-19 pandemic, self-efficacy is effectively predictive of the intention to use sports apps.*

### 2.3. Social Norms (SNs) and Sports Apps

Social norms (SNs) refer to behavioural norms that regulate interpersonal social relations, and different types of social norms play different roles in regulating such relations. According to Cialdini and Goldstein, SNs consist of subjective ones that people can perceive and external ones that people customarily follow [38]. Since RP about the COVID-19 pandemic is more attributed to subjectivity, this study mainly discusses SNs from the subjective perspective. Prior studies have demonstrated that SNs significantly influence people’s willingness to use various forms of technology [39]. Venkatesh and Davis argue that when studying the intention to use technology applications, they find that people tend to give priority to others who expect them to engage in relevant behaviours [33]. Ajzen proposes that even if people themselves disagree with an act or its consequences, they will still choose to do it if they believe that one or more important referents believe that they should and that they have sufficient capacity to comply with the referents’ requirements [40].

**Figure 1:**
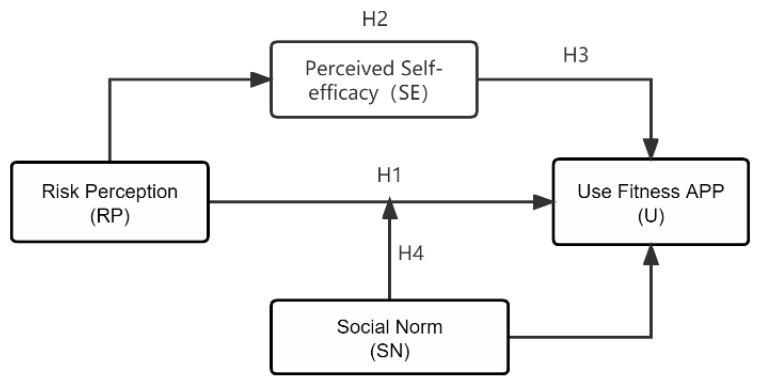
Conceptual Framework.

Health threats from public health emergencies are liable to gather individuals into a community of common interest. From the instinct of self-protection, individuals, in addition to relying on their own judgement, tend to actively seek group consensus and adopt behaviours that are more conducive to survival. Studies have shown that SNs have a noteworthy effect on people’s willingness to exercise and maintain a healthy diet when they are aware of the harms of obesity on their health [41]. In this study, it is assumed that the SNs shaped by social relations such as star coaches in sports apps, social media influencers and friends and relatives exert an impact on the individuals’ intention to use sports apps. That is, SNs formed under COVID-19 containment provide individuals with clues to explain the situation and construct the demands, and then those individuals will more likely accomplish something under the influence of such norms. Therefore, this study explores the influence of SNs as a moderating variable in the relationship between RP and usage intention and proposes the following conceptual framework (Figure 1) and hypothesis.

**H4.**  *Social norms play a moderating role in the relationship between risk perception and intention to use sports apps.*

## 3. Materials and Methods

### 3.1. Participants and Data Collection

In accordance with the requirements imposed under pandemic containment policies, this study was conducted with the assistance of a survey company in mid-January 2022 by non-contact survey collection of questionnaires. According to the “White Paper on China’s Sports and Fitness Industry Development Trends in 2019” released by iResearch in 2019, users of sports and fitness apps in second-tier cities accounted for a large proportion and involved relatively young age brackets. Despite China’s increasing Internet penetration, the percentage of people using sports APPs varies greatly by region for a variety of reasons (including age, gender, education level, economic conditions, infrastructure, etc.). Considering that the economic development of the Yangtze River Delta region of China is in a leading place in the country, citizens in this area have above-average living standards, and their pursuit of intelligent and digital life is more evident. We set a sample with high socio-economic status and digital media usage, a reference sample selected in line with the concept of demonstration area, so that the results of the influence of risk perception on the intention to use sports APPs could be illuminating and replicable for future policy development, APP use and improvement. Therefore, samples collected in this region could derive more convincing results [42]. As of 16 January 2022, 1600 participants had completed anonymous questionnaires, in which no identifiable data collection was involved. With 234 invalid questionnaires screened out, including blank and inauthentic ones, a total of 1366 valid samples were obtained, with an effective rate of 85.38%. Of these, 638 respondents were male (46.7%) and 728 were female (53.3%). Young respondents made up a large proportion, with the 26–30 age group accounting for 7.8% and 31–40 age group 61.8%. In addition, 19.6% of participants did not have a high school diploma and 51.5% had a university degree or higher. The age distribution of this sample dataset was similar to that reported by Fung Global Retail and Technology [43], which indicated that the younger generation (i.e., those in their 20 s and 30 s) make up major groups who do sports and use sports apps.

### 3.2. Measurement

This study had the intention to use sports apps as a dependent variable and risk perception (RP), social norms (SNs) and self-efficacy (SE) as independent variables. All survey items were designed according to the 5-point Likert scale with categories ranging from 1 = ‘strongly disagree’ to 5 = ‘strongly agree’. All the questions were given in the form of positive descriptions, so a higher score indicates a stronger level of intention.

### 3.3. Reliability and Validity Analysis

Reliability can be defined as the proportion of observed variance in scores, and Cronbach’s alpha (α) is the most widely used approach for the assessment of internal consistency of items involved [44]. The standard values of Cronbach’s alpha (α) for variables in this study are all verified to be greater than or equal to 0.70, indicating that the scales used herein are without exception internally consistent [45].

For PR about the pandemic (α = 0.802), three statements were set: (1) I think the pandemic is fatal; (2) The impact of the pandemic is beyond my cognitive expectations; and (3) The pandemic has generated a fear in me. Three statements were proposed with regard to the variable of SNs (α = 0.902) to explore its influence on the public’s usage intention: (1) A star trainer or an influencer in sports apps can monitor my exercise and keep me going; (2) Professional trainers and fitness influencers provide live streaming guidance for my use of sports apps; and (3) Family and friends on the sports app make me more competitive, more active and serious about working out. For the SE variable (α = 0.974), three statements were proposed: (1) I believe I could customize my diet and training by using sports APPs; (2) I believe I could record body parameters combined with wearable devices by using sports APPs; and (3) I believe I could monitor and analyse the motion data by using sports APPs. For the intention to use sports apps (α = 0.727), two statements were set: (1) I would choose to use sports apps for better outcomes in exercise during COVID-19; and (2) I would continue to use sports apps after COVID-19. An acceptable level of reliability (Cronbach’s α > 0.7) can be seen for each of the three variables that influence the intention to use sports apps.

Validity analysis can be used to test the accuracy of construct measurement, i.e., the validity of the scale. The Kaiser–Meyer–Olkin (KMO) value in this study is 0.702, and the results of the Bartlett test (X² = 3439.01, df = 21, *p* < 0.001) are significant. The KMO value for the RP variable is 0.710, for SNs 0.735, for SE 0.781 and for usage intention 0.5. These data demonstrate the validity of the scales in this study.

## 4. Data Analysis and Results

### 4.1. Descriptive Statistics

Most participants exhibited a high level of RP for COVID-19 (M = 3.713; SD = 1). They also exhibited high SE when deciding whether to use sports apps (M = 4.412, SD = 0.68), and reflected significant impressions they received from SNs (M = 4.368, SD = 0.675). These data suggest a marked positive correlation between RP, SE, SNs and usage intention (U). RP is significantly positively correlated with SNs and U, verifying H1. In addition, SPE is significantly positively correlated with U, supporting H3.

### 4.2. HLR Analysis

The data analysis of this study is divided into two phases. The first phase is aimed to analyse the association between the independent variables of risk perception, self-efficacy and social norms and the dependent variable of public intention to use sports APPs. During this stage, three analyses were conducted by hierarchical regression: the first analysis examines the relationship between demographic variables (gender, age and education level) and the dependent variable; in the second analysis, risk perception factors are added into the model to examine whether they have significant impact on the dependent variable; public self-efficacy and social norms are then entered into the regression model additionally in the last analysis to increase the explanatory power of the model. To clarify the respective explanatory power and role of the two independent variables in the third analysis (self-efficacy and social norms) in the relationship formation between risk perception and usage intention, the second phase of data analysis examines the mediating effect of self-efficacy and the moderating effect of social norms.

The first phase of this study examining demographic variables and hierarchical regression results was in line with the initial prediction of usage intention regarding sports apps during the pandemic (Table 1). In previous studies, gender variables have been an important predictor. As shown in the model here, no significant difference was found in usage intention between men and women (Male = 1, β = 008, *p* > 0.05) in using sports apps during the pandemic, suggesting that no disparity exists between the two genders in deciding whether to use such apps. No significant difference was found in relation to age either (β = −0.017, *p* > 0.05), and the demographic model generated no significant results: F (3,1363) = 0.70, *p* = 0.55, contributing a mere 0.2% in the change in usage intention. The inclusion of RP factors in the second-phase study increased the variance of behavioural intention (F (4,1362) = 24.53, *p* < 0.001), accounting for 7% of behavioural intention changes. The third phase of the study, in which the public’s SE and SNs are added to the regression model, report significant changes in R². The addition of those variables increased the interpretative power of the model, contributing 48.1% of the usage intention. SE (β = 0.214, *p* < 0.001) during the pandemic show noteworthy positive correlation with the intention to use sports apps. The third phase verifies H3.

This study correlates the perception of the pandemic with behavioural measures taken. A high degree of consistency is shown in the public’s perception of the risks posed by the pandemic: as shown in the survey results, most people believe that the pandemic has deadly consequences and generates fear, and that its impact goes beyond what they are able to perceive; accordingly, the more people perceive risk, the more they are inclined to use sports apps.

The study finds that SE has a mild mediating effect between RP and usage intention. The interaction of SE × RP is not significantly related to dependent variables, and SNs play a notable moderating role between RP and usage intention (Model 2 in Table 2), although the relation is negative (β = −0.021, *p* < 0.05).

**Figure 2:**
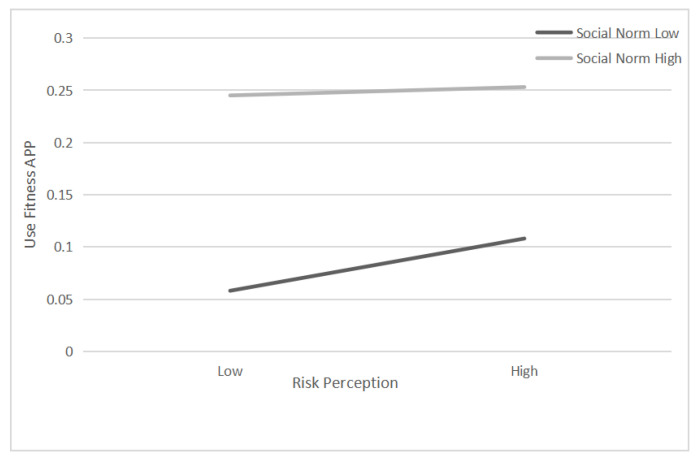
Social Norm as a function of the interaction between risk perception and use of sports apps.

To test the moderating role of people’s SE and SNs between RP about COVID-19 and the use of sports apps, this paper first uses PROCESS macro to verify the two-way interaction between the two factors and RP, and the results show that the interaction term RP × SN exerts a significant effect, while SE × RP does not. Secondly, HLR analysis was performed regarding H4 (Table 2). Model 1 uses the independent variables RP and SN as control variables, followed by the addition of the variable RP × SN in Model 2. Table 2 reveals a significant interactive effect of the two-way interaction between RP and SN on U (Model 2, RP × SN, β = −0.021, *p* < 0.01). Despite the positive correlation between RP and the intention to use sports apps, SNs have a reverse influence (β = −0.021). Regardless of the strength of SNs, RP and usage intention are positively correlated (Figure 2), attesting to the moderating role of SNs.

## 5. Discussion

During the COVID-19 pandemic, people have been constantly vigilant about the volatility of the situation, and the ever-increasing perceived risks prompt the public to adopt self-protective behaviours and actively prevent health problems caused by infection. This study used a questionnaire survey to measure RP about the coronavirus against the background of the new life state under pandemic containment in China, and to discuss the impact of the public’s RP on the intention to use sports apps. As this study has found, RP about COVID-19 not only constitutes a major factor in the public’s choices regarding the use of sports apps for self-protection [31], but also has a positive impact on the public’s intention to use such apps. In this way, the study fills the research gap with regard to the impact of RP theories on public behaviours in the context of life during global public health events.

Data in Figure 1, Table 1 and Table 2 all indicate an overall conformity between the hypotheses and survey results. The key factor influencing the intention to use sports apps is RP, as it is a key condition for motivating self-protective behaviours. Due to its high infection rate and considerable harms, among other features, the pandemic had a psychological impact on the public and strengthened their awareness of RP. Relatively high RP drives greater and more frequent sports app use. To make the public more ready to use sports apps, improve their physical health and boost their immunity, it is necessary for app research and development engineers to pay close attention to the RP psychology among people when developing or upgrading products so that they can continuously optimize functions in their technologies to meet users’ ever-changing needs. In addition, from policy formulation to information dissemination in the media, government departments also need to actively guide the public on the RP of public health events, inform the public about how they can respond to such events, and encourage them to participate more in physical exercise and actively use digital technologies to improve the outcomes of workouts.

Further, the study reveals a significant positive correlation between self-efficacy and usage intention, proving that SE is of great importance. Therefore, while the importance of RP is emphasized, the positive influence of SE on the intention to use sports apps should not be ignored. Meanwhile, SNs play a noticeable moderating role between RP and the intention to use sports apps. Therefore, it is desirable to promote the active use of social networks and community relationships to strengthen SNs, improve public health awareness and promote people’s willingness to use sports apps.

Based on the investigation of the COVID-19 pandemic, sports apps will become important practical tools in the field of sports and fitness. This form of exercise can minimize contact and social interaction, which is important during a pandemic. While this study is of practical significance, it also proposes requirements on future development of sports apps whereby multiple factors including technology, policy and capital should form a synergy, constantly improving public trust to stimulate usage intentions, constantly reducing the complexity of online technology applications to meet the needs of users to maintain PA, and constantly enhancing both physical and mental health so that society is able to respond to future public health emergencies.

## 6. Limitations

The sample collection of this study is to a large extent limited to the Yangtze River Delta (Jiangsu, Zhejiang and Shanghai), and may fail to represent all the attributes that match the demographics of present-day China; hence the limitations on the universality of the results concluded herein. Thus, it is advised that future studies include more cross-regional and representative samples. This study reports no significant correlation between demographic control variables, such as gender, age, and educational level, and the variable studied. This needs to be further investigated in future studies.

## 7. Conclusions

Based on risk perception theories, this study explores the influence of risk perception, self-efficacy and social norms on people’s intention to use sports apps and finds that the three elements are all significantly and positively related to the usage intention in question. This study is distinguished from previous studies in that it takes the particular social situation under COVID-19 containment as the context, is conducted from the perspective of risk perception of public health emergencies, and identifies the influence of the Chinese public’s risk perception on the intention to use sports apps. In this way, it expands the research scope regarding sports apps, addresses the research gap concerning sports apps in the context of the special social situation brought about by global public health events, and thus provides reference for research on risk perception of the pandemic in other countries, in essence enriching the scope of risk perception theories. It is notable that the future of humankind is subjected to the constraint of a variety of unpredictable factors such as epidemics, natural disasters and wars, and thus likely to see the emergence of unknown social forms so that new kinds of risk perception will also arise. The discussion in this paper considers the newly formed ‘online + offline’ social forms caused by policies of closure, quarantine and goals towards zero COVID-19 transmissions outside quarantine areas. In this sense, it provides researchers with a special perspective to study social forms that may be confronted in the future, characteristic of China but illuminating to wide areas of the world. ‘To be or not to be’ has always been one of the core essentials of humanities. Research on new social forms and new lifestyles has not only theoretical value in the scope of human sociology, but also practical significance for future forms of human existence in that it serves to ‘examine the present to shape the future’.

## Figures and Tables

**Table 1:** Multiple linear regression of intention to use sports apps.

Step	Variable Entered	β	β	β
1	Gender (male = 1)	−0.008	−0.014	0.011
	Age	−0.017	−0.019	−0.000
	Education	0.008	0.019	−0.007
2	RP		0.111 ***	0.022 ***
3	SN			0.214 ***
	SE			0.230 ***
	N	1366	1366	1366
	R²/adjusted R^2^	0.01/0.00	0.07/0.07	0.48/0.48
	ΔR^2^	0.00	0.07	0.41
	ΔF	0.703	95.862 ***	541.533 ***
	Model F	0.703	24.530 ***	209.863 ***

Note: *** *p* < 0.001.

**Table 2:** Moderated regression analyses of SN.

Mode	Variables	Standardized Coefficients	R²	Change Statistics		Collinearity Statistics	
				∆R^2^	∆F	Tolerance	VIF
1	RP	0.026	0.458	0.458 **	575.869	0.913	1.096
	SN	0.422				0.913	1.096
2	RP	0.120	0.460	0.002 **	4.439	0.880	1.137
	SN	0.487				0.779	1.283
	RP × SN	−0.021				0.850	1.177

Note: ** *p* < 0.01.

## Data Availability

The data presented in this study are openly available by contacting the corresponding Author.

## Abbreviations

PA	physical activity
RP	risk perception
SE	self-efficacy
SNs	social norms
U	usage intention
HLR	hierarchical linear regression
